# Distinguishing four components underlying physical activity: a new approach to using physical activity questionnaire data in old age

**DOI:** 10.1186/1471-2318-10-20

**Published:** 2010-05-03

**Authors:** Lisanne M Verweij, Natasja M van Schoor, Joost Dekker, Marjolein Visser

**Affiliations:** 1 EMGO Institute for Health and Care Research, VU University Medical Center, Amsterdam, the Netherlands; 2 Department of Rehabilitation Medicine, VU University Medical Center, Amsterdam, the Netherlands; 3 Institute of Health Sciences, Faculty of Earth and Life Sciences, VU University, Amsterdam, the Netherlands

## Abstract

**Background:**

It is evident that physical activity has many benefits, but it often remains unclear which types of activity are optimal for health and functioning in old age. The aim of this methodological study was to propose a method for distinguishing four components underlying self reported physical activity of older adults: intensity, muscle strength, turning actions and mechanical strain.

**Methods:**

Physical activity was assessed by the validated LAPAQ questionnaire among 1699 older adults of the Longitudinal Aging Study Amsterdam. Based on expert consultation and literature review, the four component scores for several individual daily and sports activities were developed. Factor analysis was performed to confirm whether the developed components indeed measured different constructs of physical activity.

**Results:**

Based on the factor analyses, three components were distinguished: 1. intensity and muscle strength loaded on the same factor, 2. mechanical strain and 3. turning actions. Analyses in gender, age and activity level subgroups consistently distinguished three factors.

**Conclusion:**

Future research using these components may contribute to our understanding of how specific daily and sports activities may have a different influence on health and physical functioning in old age.

## Background

It is well established that physical activity is essential in healthy aging. With aging, fitness levels decrease and functional impairments and chronic diseases often arise [1]. These health declines may lead to functional limitations and can result in an increased risk of falling and a diminished independence among older adults [2]. By engaging in physical activity, these age related declines can be reduced and even improvement of neuromuscular and physical function, quality of life and disease risk in older adults may occur [3-13].

Recently, new physical activity recommendations for older adults were published based on the latest evidence and expert consensus [1]. It was stated that physical activity of older adults should emphasize moderate-intensity aerobic activity, muscle strengthening activities and possibly weight bearing activities. However, it remains unclear what type of activities are optimal for improving health and preventing disease in old age [14]. The following example illustrates the complex influence of physical activity on health. During tennis, the high weight bearing forces may be beneficial for maintaining bone density in postmenopausal women [15] but may increase the risk for osteoarthritis [16]. Additionally, the many turning actions during tennis may increase the risk of falling [17], but the high intensity and muscle strength involved may prevent injury and reduce the risk of cardio-vascular disease [18,19]. This example illustrates that a single activity consists of different components, which all may have different relationships with a health outcome.

The aim of the present methodological study is to suggest a new way of evaluating physical activity of older adults by proposing different components underlying self-reported physical activity, namely intensity, muscle strength, turning actions and mechanical strain. The study is based on data from a physical activity questionnaire, as currently available objective methods to assess physical activity level do not provide information on the type of the activities performed. In distinguishing between these four underlying components new insights in the relationship between physical activities and prevalent and incident disease and functioning may be gained, from which a more tailored physical activity advice for older persons can be developed in future studies.

## Methods

### Subjects

Data were used from the Longitudinal Aging Study Amsterdam (LASA), an ongoing study in a representative sample of 3107 older persons, aged 55-85 years. Respondents were recruited from three areas in the Netherlands and were stratified for age, gender and 5-year mortality at baseline (1992/1993). Measurements and interviews on social, cognitive, psychological and physical functioning are repeated every three years [20]. For the present study, data were used from respondents who completed the third data collection cycle of LASA (1998/1999, n = 1874). Participants were excluded when they were bedridden, wheelchair dependent or had a missing mobility status (n = 149) and when they performed no daily or sport activities (n = 26), resulting in 1699 respondents for analyses. The LASA study was approved by the Ethical Review Board of the VU University Medical Center. All respondents signed informed consent on entering the study.

### Measurements

Daily and sport activities were assessed using the LASA Physical Activity Questionnaire (LAPAQ), which provides a valid and reliable measure of physical activity in older adults [21]. Frequency and duration of activities over the past 2 weeks were asked for walking, bicycling, light and heavy household work and a maximum of two sports.

The variables gender, age, education and body mass index were used to describe the study sample. Education was divided into three categories; low (elementary school or less), medium (primary and secondary education) and high education (college and university). Body mass index (BMI) was calculated using measured weight and height and was expressed in kg/m^2^.

### Development of component scores

Based on literature review and expert consultation, every LAPAQ activity was given a score for the intensity, the required muscle strength, the mechanical strain and the turning actions component. All experts (three occupational therapists and three professors in the area of biomechanics, allied health care and healthy aging) individually decided on the scores. The proposed scores were discussed by the three professors to reach consensus. Scores and rationale were provided by other studies for the intensity, mechanical strain and turning action component scores [22-24]. Because no score was available for the muscle strength component we constructed and validated this component for the purpose of this study.

#### Intensity

The intensity of physical activities was expressed in metabolic equivalents (MET) as described in the compendium of Ainsworth, a reliable classification system [25]. The compendium contains a coding scheme to classify activities according to their intensity, with one MET being equivalent to 1 kcal*kg body weight*hour. In the present study, MET scores were adapted for older persons to adjust for the fact that older persons generally perform these activities at a lower intensity [26].

#### Mechanical strain

The mechanical strain scores represent the loading of the bone and were derived from ground reaction forces (GRF) expressed in multiples of body weight [27]. A score of 1 was given to activities with GRF values up to one time the body weight (non weight bearing activities and standing activities). Score 2 represented those activities with GRF values between one and two times body weight (weight bearing activities). Score 3 described those activities with GRF values between two and four times body weight (activities including explosive actions such as sprinting and running) and score 4 indicated physical activities with GRF greater than four times body weight (activities including jumping). This score has a good face validity [23].

#### Turning actions

In the original mechanical strain classification by Groothausen et al. turning actions were incorporated in the mechanical strain score 3 (activities including sprinting and running) [24]. However, as Besier et al. noted, turning actions are also present in jumping, squatting and weight bearing activities [28]. Therefore, the turning actions component was separated from the mechanical strain component. Turning actions were defined as activities in which transversal rotations of the lower extremities were present. The turning action scores ranged from 1 to 3, with the highest score representing activities associated with numerous turning actions, such as dancing and tennis, and the lowest score indicating few to no turning actions during the activities, such as walking and bicycling.

#### Muscle strength

The muscle strength component indicates the amount of required lower body muscle strength to perform an activity. Literature review did not provide information on strength required during specific activities. Therefore, the muscle strength associated with specific activities was based on expert consultation. This resulted in the following scores: score 4 contained activities involving jumping and squatting actions; score 3 represented activities which include running or cycling; score 2 included activities related to walking; score 1 included standing and sitting activities. The validity of this score was examined by assuming that those who frequently perform activities requiring a high muscle strength would have a higher quadriceps and hand grip strength as assessed by dynamometry. Quadriceps muscle strength is representative for lower extremity strength which is extremely important for activities of daily living [2], while handgrip strength is a more general measure for overall muscle strength [29].

We used data from a sub sample of 418 respondents of LASA (1999/2000) with quadriceps strength and hand grip strength measurements [30]. Quadriceps muscle strength was measured as the maximal leg extension strength, using a hand held dynamometer (MicroFET, Hoggan Health Industries Inc., Draper, UT) according to the method of Hsieh and Philips [31]. Strength measurements were performed on both legs at least four times, with a maximum of seven times. The maximum strength measurements of both legs were added and divided by two. Handgrip strength was measured with a strain-gauged dynamometer (Takei TKK 5001; Takei Scientific Instruments, Tokyo, Japan). Two maximum force measurements were conducted with each hand. Again, the maximum strength measurements of both hands were summed and divided by two.

#### Mean component score per person

Based on the individual activities performed by a person and the component scores for those activities, a mean score for each component was calculated for that person [23]. For example, a respondent who performed light household activities (2.5 MET) and played tennis (6.0 MET) would receive a mean intensity component score of 4.3 MET.

### Statistical analyses

Analyses were performed using SPSS, version 14.0 (Chicago; SPSS Inc). P values less than 0.05 were considered statistically significant. First, the validity of the developed muscle strength component score was assessed in the subsample by concurrent and construct validity. A Spearman's correlation coefficient of at least 0.30 between the developed muscle strength component score and actual quadriceps and handgrip strength measurements would indicate medium concurrent validity [32]. The correlations of the actual strength measurements with the intensity, mechanical strain and turning action component scores would have to be lower to indicate construct validity of the muscle strength component.

Secondly, we assessed whether the four developed component scores indeed measured different constructs of physical activity. A principal component analysis with Varimax rotation was performed in the total study sample. In determining the number of factors which can be distinguished, the eigenvalues, the explained variance, intercorrelations and the scree test were visually inspected. Although an eigenvalue larger than 1 is the most common used cut-off point in factor analyses, it is strongly recommended not to use this as the sole cut-off criteria for determining the number of factors [33]. Because only four variables were investigated the Joliffe criterion was used, excluding all components with eigenvalues under 0.7 [34]. The total explained variance was expected to be over 80% and the correlation matrix was expected to show correlations under 0.70. The scree test was inspected for breaks in the distribution [35]. To examine the robustness of our approach, the factor analyses were repeated for the following subgroups: men and women, young old (aged<73 y) and old old (aged ≥ 73 y), and inactive (<139 minutes per day) and active respondents (≥ 139 minutes per day) using the sample median as the cut point.

## Results

Descriptive characteristics of the study samples are shown in Table 1. Overall, the average age was 78 years in the sub sample and 73 years in the total sample, and respondents in both samples were physically active for about two and a half hours a day. Excluded respondents in the total samples were older, had a lower muscle strength, education level and BMI, and were less active (p < 0.05).

**Table 1:** Respondent characteristics of the study sample and the validation sub sample.

Characteristics	Sub sample (N = 418)	Total sample (N = 1699)
Age (years, SD)	78 (6.1)	73 (8.0)
Gender (% female)	54	54
Education (%)		
Primary	37	38
Secondary	53	50
College/University	10	13
BMI (kg/m^2^, SD)	27.2 (4.1)	27.5 (4.2)
Quadriceps strength (N, SD)	146 (40)	-
Handgrip strength (kgf, SD)	27.9 (10.2)	25.6 (10.6)
Physical activity (min/day, SD)	155 (117)	157 (102)

The final component scores per activity are presented in Table 2. The mean component scores (standard deviation) for the total study sample were 3.60 (0.46) for intensity, 1.49 (0.21) for mechanical strain, 1.55 (0.21) for turning actions and 2.54 (0.35) for muscle strength.

**Table 2:** The four developed physical activity component scores for the individual activities included in the LASA Physical Activity Questionnaire (LAPAQ).

LAPAQ Activity	Intensity component score	Mechanical strain component score	Turning actions component score	Strength component score
**General:**				
Walking outdoors	3.5	2	1	2
Bicycling	4.5	1	1	3
Light household	2.5	1	2	2
Heavy household	4.5	2	2	4
**Sports:**				
1. Distance walking	4.0	2	1	3
2. Distance cycling	6.0	1	1	4
3. Gym/Game	4.0	2	2	2
4. Home trainer	4.0	1	1	2
5. Swimming	5.0	1	2	3
6.(Folk) Dancing	5.0	3	3	2
7. Bowling/Jeu de Boules	3.5	2	2	3
8. Tennis/Badminton	6.0	3	3	3
9. Jogging/Running/Speed walking	6.0	3	1	3
10. Rowing	5.5	1	1	3
11. Sailing	3.0	1	1	2
12. Billiards	2.5	1	1	3
13. Fishing	3.0	1	1	1
14. Soccer/Basketball/ Hockey	6.0	4	3	4
15. Volleyball/Baseball	5.0	4	3	4
16. Skiing	6.0	2	3	4
17. All other activities	4.0	2	2	2

Concurrent and construct validity of the muscle strength component score were investigated using measured quadriceps and handgrip strength (Tables 3 and 4). Positive correlations between the strength component score and measured quadriceps and handgrip strength were found (r = 0.36 and r = 0.37 respectively) indicating medium concurrent validity. However, the intensity component score correlated equally high with measured quadriceps strength and handgrip strength (r = 0.33 and r = 0.32 respectively) indicating poor construct validity. A strong correlation was found between the muscle strength component score and the intensity component score (r = 0.67), but the correlation with mechanical strain (r = 0.02) and with turning actions (r = -0.25) was low.

**Table 3:** Spearman correlations coefficient between measured muscle strength and the four physical activity component scores in the sub sample.

	Measured quadriceps strength	Measured handgrip strength
Muscle strength component	.36	.37
Intensity component	.33	.32
Mechanical strain component	.02	.00
Turning actions component	-.15	-.29

**Table 4:** Intercorrelation coefficients between the four physical activity components score in the sub sample.

	Intensity component	Mechanical strain component	Turning actions component
Muscle strength component	.67	-.06	-.24
Intensity component		.02	-.25
Mechanical strain component			.31

The factor analyses identified three factors with a total explained variance of 96%: 1. intensity and muscle strength loaded on the same factor, 2. mechanical strain and 3. turning actions (Table 5). Three factors could also be identified when viewing the scree plot. The correlation matrix showed correlations under 0.70. In repeating the factor analyses within gender, age and activity level subgroups, the same three factors were consistently distinguished showing less than 10% change (data not shown). Results of additional factor analyses suggest that mean scores are indeed more important than sum and maximum scores (data not shown).

**Table 5:** Factor analysis based on the four physical activity component scores.

	Factor			
	1	2	3	4
Item*				
Muscle strength component	.96	-	-	-
Intensity component	.90	-	-	-
Mechanical strain component	-	.99	-	-
Turning actions component	-	-	.99	-
**Value**				
Eigenvalue	2.02	0.98	0.84	0.15
% Variance Explained	51	25	21	4

* Only factor loadings >0.4 are shown.

## Discussion

The aim of this methodological study was to propose a new approach to use physical activity data of older persons obtained by questionnaire. Four underlying components of physical activity were distinguished based on literature and expert consultation, including intensity, muscle strength, mechanical strain and turning action components. Results from the factor analyses showed that the mechanical strain and turning actions components indeed were distinct factors, but intensity and muscle strength loaded onto a single factor. Therefore, three underlying components of physical activity could be distinguished. Using these components in future research may help our understanding of the association between physical activity and health in old age, which will ultimately lead to a more specific and tailored physical activity advice for older persons.

The intensity, mechanical strain and turning action component scores were based on previously developed scores which were slightly adapted [22,27]. The muscle strength component score was developed and validated against actual measurements of muscle strength. Although these concepts are clearly related, it should be kept in mind that the questionnaire asks what a person does while the strength measure reflects what a person is able to do. The results showed that a high strength component score was indeed correlated with higher values of quadriceps strength and hand grip strength. However, construct validity was limited because of the high correlation of the muscle strength component score as well as the intensity component score with measured muscle strength. This was confirmed by the results of the factor analysis, showing that the intensity component and the muscle strength component loaded on a single factor. Additional factor analyses in different subgroups of older adults consistently confirmed this.

To confirm whether the four proposed components indeed each represented a different aspect of physical activity, we performed a factor analysis. These results suggest that the intensity and muscle strength components as developed in this study measure the same underlying construct and that mechanical strain component and the turning actions component were to distinctive, additional factors. This implies that future analyses should contain either the muscle strength component or the intensity component, or a combined average score.

Some limitations of this study must be reported. Our main aim of this study was to propose a new conceptual use of physical activity questionnaire data. The generalizability of our findings to other physical activity questionnaires and to different populations is not known. Therefore, future studies are needed to validate our approach and underlying assumptions in other datasets using questionnaires that contain the same domains as the LAPAQ (walking, bicycling, household activities and sports) and to extend our results using questionnaires that include other domains. Second, the total amount of physical activity performed by the respondents, expressed in minutes per day, or the total time spend on each activity was not incorporated in each persons' component score. As our components are based on the nature of activities (which type of activities are similar in their component score) we feel it is more applicable to keep nature and duration separated, because scores are otherwise more difficult to make, and to interpret. Nevertheless, when relating the component scores to health outcomes in future studies, adjustment for total activity level or activity duration should be made [36]. Third, in this type of studies it would also be of interest to investigate potential interaction between components. For example, it should be examined whether persons with a high mechanical strain component score could attenuate their risk for knee OA by having a high muscle strength component score. Moreover, both observational and intervention studies are needed to further elucidate the potential role of different types of physical activity in the development of knee OA to allow more specific physical activity advice for older persons. Fourth, future studies should ask all sports a person performs. Our physical activity scores may be underestimated as the physical activity questions were limited to the most usual daily activities and a maximum of the two most intensive sports activities. Finally, further research is needed to translate the current results to clinical practice. Although the LAPAQ can be completed in approximately six minutes and is easy to fill in (0.5% of the respondents had missing values [21]), this is too long for the clinical setting. Seven similar questionnaires were investigated and were also found too long and complex for routine administration, because all questionnaires were developed for the research setting [37]. Thus, simple scoring lists including the most relevant types of activities should be developed and easy interpretation of the results by the clinician is needed to allow for good feasibility and interpretation in a clinical setting.

## Conclusion

The results of this study show that different components underlying physical activity in older adults can be identified. The results suggest that at least three components may exist including a mechanical strain component, a turning actions component and a combined intensity/muscle strength component. As recent studies by our group [38,39] and others [40-44] show, these components may differ in their relation with health outcomes.

The use of these components in future studies may further improve our understanding of the mechanisms by which physical activities influence prevalent and incident disease in old age, ultimately enabling the development of specific and tailored physical activity advice for older adults.

## Competing interests

The authors declare that they have no competing interests.

## Authors' contributions

LV and MV had a role in the study concept and design. MV and NvS provided acquisition of subjects and data. LV, NvS, JD, MV contributed to the analysis and interpretation of data and preparation of the manuscript. All authors read and approved the final manuscript.

## Pre-publication history

The pre-publication history for this paper can be accessed here:

http://www.biomedcentral.com/1471-2318/10/20/prepub
